# Structural basis for the inhibition of PRC2 by active transcription histone posttranslational modifications

**DOI:** 10.1038/s41594-024-01452-x

**Published:** 2025-01-07

**Authors:** Trinity Cookis, Alexandria Lydecker, Paul Sauer, Vignesh Kasinath, Eva Nogales

**Affiliations:** 1 Department of Molecular and Cell Biology, University of California, Berkeley, CA USA; 2 California Institute for Quantitative Biosciences (QB3), University of California, Berkeley, CA USA; 3 Molecular Biophysics and Integrative Bio-Imaging Division, Lawrence Berkeley National Laboratory, Berkeley, CA USA; 4 Department of Biochemistry, University of Colorado, Boulder, CO USA; 5 Howard Hughes Medical Institute, University of California, Berkeley, CA USA

**Keywords:** Cryoelectron microscopy, Enzyme mechanisms, Gene silencing, Histone post-translational modifications

## Abstract

Polycomb repressive complex 2 (PRC2) trimethylates histone H3 on K27 (H3K27me3) leading to gene silencing that is essential for embryonic development and maintenance of cell identity. PRC2 is regulated by protein cofactors and their crosstalk with histone modifications. Trimethylated histone H3 on K4 (H3K4me3) and K36 (H3K36me3) localize to sites of active transcription and inhibit PRC2 activity through unknown mechanisms. Using cryo-electron microscopy, we reveal that histone H3 tails containing H3K36me3 engage poorly with PRC2 and preclude its effective interaction with chromatin, while H3K4me3 binds to the allosteric site in the EED subunit, acting as an antagonist that competes with activators required for spreading of the H3K27me3 repressive mark. Thus, the location of the H3K4me3 and H3K36me3 modifications along the H3 tail allows them to target two requirements for efficient trimethylation of H3K27 by PRC2. We further show that the JARID2 cofactor modulates PRC2 activity in the presence of these histone modifications.

## Abstract (web summary)

Structures reveal that histone H3K36me3 and H3K4me3 modifications reduce Polycomb repressive complex 2 (PRC2) function through the inhibition of histone tail engagement and antagonistic binding to the allosteric site, two important requirements for the efficient trimethylation of H3K27 by PRC2.

## Main

The dynamic recognition, deposition and removal of posttranslational modifications (PTMs) on histone proteins facilitate the establishment of specific gene expression programs. Many chromatin-modifying enzymes are large protein complexes with catalytic and regulatory regions capable of sensing the chromatin environment. Preexisting chromatin marks may act as recruitment platforms and/or directly stimulate or restrict the catalytic activities of chromatin-modifying enzymes. The precise recruitment, activation and interplay between the chromatin-modifying machinery and the chromatin state are vital to define active or repressed gene states during development and for maintaining them throughout the organism’s lifespan.

Polycomb repressive complex 2 (PRC2) is an essential epigenetic regulator that marks genes for repression through its deposition of trimethylation on histone H3 at K27 (H3K27me3)^1^. It contains four core subunits (EZH1/2, EED, RBAP46/48 and SUZ12) and additionally associates with accessory proteins that impact the recruitment and catalytic functions of the complex: AEBP2 and JARID2 in PRC2.2 and EPOP/PALI1 and PHF1/MTF2/PHF19 in PRC2.1 (refs. ^2,3^). The interaction of PRC2 with substrate nucleosomes involves mainly two structural elements within the catalytic subunit EZH2, the bridge helix and the CXC domain. These elements interact with nucleosomal DNA through electrostatic interactions and contribute to extensive contacts that help guide the histone H3 tail into the active site^4,5^.

PRC2 activity is regulated on chromatin through crosstalk with several histone PTMs. A central mechanism of PRC2 activation is the recognition of PRC2-trimethylated lysine peptides by its EED subunit, resulting in conformational changes within PRC2 that lead to the allosteric activation of the complex^4,6,7^. As a consequence, H3K27me3, the product of PRC2 enzymatic activity on nucleosomes, binds to the EED and activates EZH2, thus enabling the spreading of this modification across chromatin to establish and maintain repressed domains^4,7–10^. PRC2 also trimethylates the accessory proteins JARID2 (at K116) and PALI1 (at K1241), which can then mimic H3K27me3 to serve as allosteric activators of the complex^6,11–14^. Recognition of allosteric activators at the EED regulatory site results in the folding of the stimulatory response motif (SRM) helix, which interacts with the catalytic SET domain of EZH2, enhancing its enzymatic activity^4,7,10^. PRC2.2 accessory proteins, JARID2 and AEBP2, additionally mediate interactions with chromatin previously modified by PRC1 with H2AK119Ub to further promote PRC2 activity at defined genomic locations^5,15,16^.

Despite the accumulation of molecular details depicting interactions between PRC2 and chromatin in states that promote catalytic activity^5,17–19^, there is still a poor understanding of the mechanisms proposed to exclude PRC2 activity from actively transcribed chromatin, such as RNA^20–24^ and histone PTMs^25,26^. The H3K4me3 and H3K36me3 histone modifications localize at promoters^27–31^ and gene bodies^28,30,32,33^ of actively transcribed genes, respectively. Genomic locations harboring these modifications are practically devoid of H3K27me3 (refs. ^34–36^) and in vitro biochemical assays have demonstrated that both H3K4me3 and H3K36me3 directly inhibit PRC2 enzymatic activity^5,18,25,26^. It has also been shown that JARID2 can enhance the activity of PRC2 in the presence of both H3K4me3 and H3K36me3 chromatin modifications^5,18,37^. Deciphering the crosstalk among PRC2, its accessory proteins and the epigenetic landscape is required to understand how the activities of Polycomb proteins are regulated to control gene repression and establish heterochromatin boundaries.

Here, we used cryo-electron microscopy (cryo-EM) to determine structures of PRC2.2 (from here on, PRC2) complexes engaged with nucleosome substrates containing H3K4me3 or H3K36me3, both in the presence and in the absence of the methylated JARID2 K116. We discovered that H3K4me3-containing and H3K36me3-containing nucleosomes inhibit PRC2 using two distinct mechanisms because of the differences in their positions along the histone H3 tail. These mechanisms of inhibition target two important requirements for the efficient trimethylation of histone H3K27 and support a model in which chromatin regions rich in H3K4me3 or H3K36me3 can act as boundaries to restrict PRC2 function to confined locations in the genome. We also further define important functions for the accessory protein JARID2 in alleviating inhibition by these chromatin modifications that may be critical when large sections of the genome require silencing during embryonic development.

## Results

### H3K36me3 reduces PRC2 engagement with the histone H3 tail

Histone H3K36me3 decorates gene bodies and interacts directly with RNA polymerase II to regulate transcription elongation^32,38,39^. The deposition of H3K27me3 is prevented at actively transcribed genes through uncharacterized mechanisms and, interestingly, histone H3K36me3 directly inhibits the activity of PRC2 in biochemical assays^5,18,25,26^. Previous cryo-EM structures of PRC2 bound to unmodified nucleosome substrates described the extensive network of interactions that facilitate the placement of K27 at the EZH2 active site for catalysis. The unmodified histone H3K36 was hypothesized to be important for PRC2 activity because of its prominent location at the entry site of the H3 tail binding channel, where it forms electrostatic interactions with the nucleosomal DNA and polar contacts with the CXC domain^5,18^. On the other hand, the PRC2 core has been shown to efficiently monomethylate H3K27 (H3K27me1) in H3K36me3 nucleosomes and, in the presence of JARID2, exhibits some level of higher-order methylation on such substrates^5,18,25,26^.

**Fig. 1:**
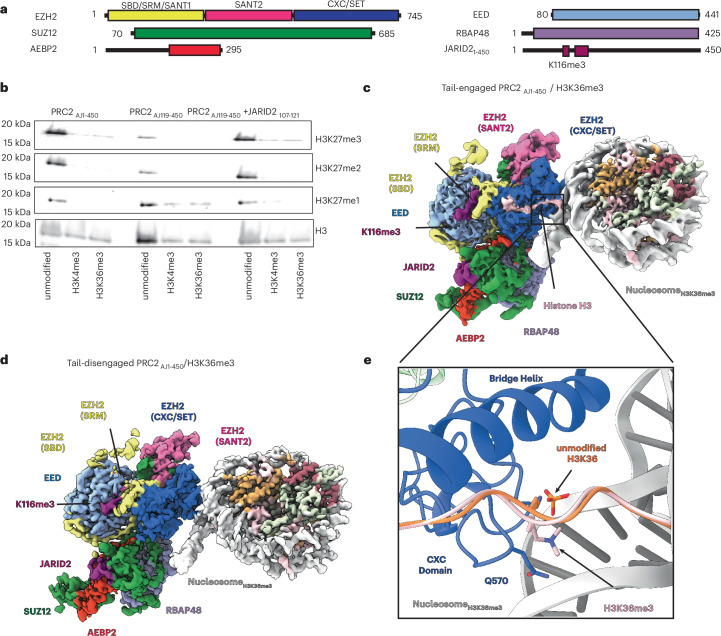
Cryo-EM structures of PRC2_AJ1__–__450_ bound to H3K36me3-modified nucleosomes. **a**, Schematic representation of protein domains in the PRC2–AEBP2–JARID2 complex used in this work, containing either JARID2_1__–__450_ or JARID2_119_. **b**, Representative methyltransferase assays performed on mononucleosome substrates that were either unmodified, H3K4me3 modified or H3K36me3 modified. Assays were repeated in triplicate with PRC2_AJ1__–__450_, PRC2_AJ119__–__450_ or PRC2_AJ119__–__450_ in the presence of 150 µM methylated JARID2 peptide including residues 107–121. **c**, Cryo-EM structure of PRC2_AJ1__–__450_ bound to an H3K36me3-modified nucleosome in which the H3 tail is engaged by EZH2 and PRC2 is in an allosterically stimulated state. **d**, Cryo-EM structure of PRC2_AJ1__–__450_ bound to H3K36me3-modified nucleosomes in which the H3 tail is not engaged by EZH2. The structures shown in **c**,**d** coexist in the sample. **e**, Comparison of the position of K36 with respect to the nucleosomal DNA in structures of PRC2 bound to unmodified H3K36 (shown in orange; PDB 6WKR) and H3K36me3-modified nucleosomes (shown in pink). Source data

To investigate the molecular basis for the inhibition of PRC2 by chromatin marked with H3K36me3, we sought to determine structures of PRC2 bound to modified nucleosome substrates using a PRC2 complex containing AEBP2 and a construct of JARID2 that included the allosterically activating K116me3 segment (referred to as PRC2_AJ1__–__450_) (Fig. 1a). As previously shown^5^, this PRC2 complex exhibits substantially reduced activity on H3K36me3-modified nucleosomes with respect to unmodified nucleosome substrates^5^ (Fig. 1b). All structural efforts described in this paper used streptavidin affinity grids^40,41^, which provided a robust sample preparation strategy to protect the PRC2–nucleosome complexes from the adverse effect of the air–water interface and enrich for nucleosome-bound PRC2 (ref. ^5^).

**Extended Data Fig. 1:**
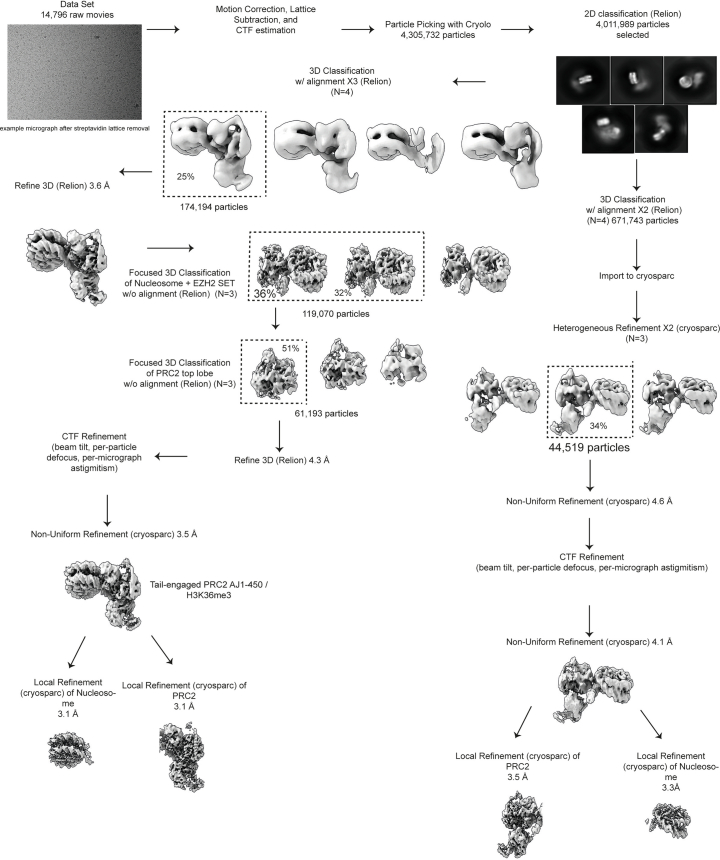
Processing workflow for PRC2_AJ1-450_ bound to H3K36me3 nucleosomes. Data collected for PRC2_AJ1-450_ bound to H3K36me3 nucleosomes was initially processed in RELION. Focused classification around the nucleosome and EZH2 SET domain resulted in two classes, one showing the histone H3 tail engaged and one lacking density for the histone H3 tail. We further refined particles with the tail engaged and performed focused classification around the PRC2 top lobe to obtain a 3.1 Å reconstruction that resolved the histone H3K36me3 side chain (left side of processing diagram). We then used the initial 4,011,989 particles obtained from 2D classification to perform 3D classification and heterogeneous refinement to select particles lacking density for the histone H3 tail followed by homogeneous and local refinement using CryoSPARC to resolve the 3.5 Å reconstruction of the ‘tail-disengaged’ state (right side of processing diagram).

**Extended Data Fig. 2:**
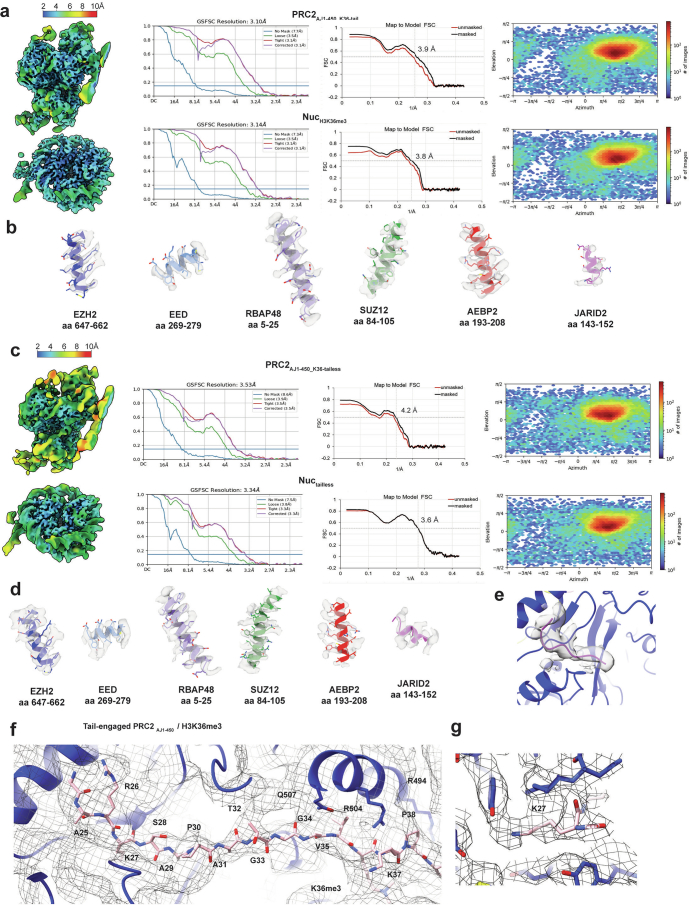
Local resolution mapped onto cryo-EM maps of PRC2_AJ1-450_ bound to H3K36me3-containing nucleosomes obtained from local refinements using masks surrounding PRC2 and nucleosome regions. For each map, the resolution at FSC = 0.143 is provided, while the map-to-model FSC plots show the masked resolution at FSC = 0.5. Each map has been locally filtered using the local resolution estimate. Angular distribution plot for particles that make up the final reconstructions are also included. **A)** PRC2_AJ1-450_ / H3K36me3 ‘tail-engaged’ state **B)** Examples of fitting of protein amino acid side chains into the density map for each subunit within the PRC2 complex. **C)** PRC2_AJ1-450_ / H3K36me3 ‘tail-disengaged’ state **D)** Examples of fitting of protein amino acid side chains into the density map for each subunit within the PRC2 complex **E)** Close up view of active site density observed in the PRC2_AJ1-450_ / H3K36me3 ‘tail-disengaged’ state corresponding to either JARID2 or the histone H3 tail. JARID2 residues 23-29 (magenta) are shown docked into the residual density but were excluded from the deposited coordinates. **F)** Cryo-EM density shown for the histone H3 tail (pink) bound to the EZH2 SET domain (blue) in the PRC2_AJ1-450_ / H3K36me3 ‘tail-engaged’ state. **G)** Close up view of histone H3K27 (pink) bound to the active site of EZH2 (blue).

Analysis of the cryo-EM data of PRC2_AJ1–450_ bound to the H3K36me3-modified nucleosome (Table 1) resolved two distinct states at 3.1-Å and 3.5-Å resolution, respectively. In the first state, the H3 tail can be seen binding to EZH2 and entering the EZH2 active site (referred to as ‘tail-engaged’) (Fig. 1c and Extended Data Figs. 1 and 2a,b), while, in the second state, density for the H3 tail appears absent (referred to as ‘tail-disengaged’) (Fig. 1d and Extended Data Figs. 1 and Extended Data Fig. 2c,d). In both the tail-engaged and the tail-disengaged states, we observe JARID2_K116me3_ bound at the allosteric site on EED. Consequently, as previously observed for activated forms of the complex, the SRM is folded and interacting with the SET-I helix of the active site, and the SANT-binding domain (SBD) is in a bent conformation (another conformational feature associated with an active state^6,7,11^) (Fig. 1c,d). The presence of H3K36me3, therefore, does not disrupt the allosteric communication between the EED and EZH2 core subunits.

**Table 1:** Cryo-EM data collection, refinement and validation statistics

	PRC2_AJ1__–__450_K36-tail_ (EMD-43361, PDB 8VNV)	Nuc_H3K36me3_ (EMD-43373, PDB 8VOB)	PRC2_AJ1__–__450_K36-tail-disengaged_, (EMD-43362, PDB 8VNZ)	Nuc_tailess_, (EMD-43363, PDB 8VO0)
**Data collection and processing**				
Magnification	×81,000	×81,000	×81,000	×81,000
Voltage (kV)	300	300	300	300
Electron exposure (e^−^ per Å^2^)	50	50	50	50
Defocus range (μm)	−0.8 to −1.8	−0.8 to −1.8	−0.8 to −1.8	−0.8 to −1.8
Pixel size (Å)	0.525	0.525	0.525	0.525
Symmetry imposed	*C1*	*C1*	*C1*	*C1*
Initial particle images (no.)	4,011,989	4,011,989	4,011,989	4,011,989
Final particle images (no.)	61,193	61,193	44,519	44,519
Map resolution (Å)	3.1	3.14	3.5	3.3
FSC threshold	0.143	0.143	0.143	0.143
Map resolution range (Å)	2.8–6	2.9–6	2.9–7	3.1–6
**Refinement**				
Initial model used (PDB code)	6WKR	6WKR	6WKR	6WKR
Model resolution (Å)	3.9	3.9	4.2	3.6
FSC threshold	0.5	0.5	0.5	0.5
Map sharpening *B* factor (Å^2^)	63	63	59	62
Model composition				
Nonhydrogen atoms	15,927	12,550	14,771	12,217
Protein residues	1,984	777	1,962	734
Ligands	49	314	0	314
*B* factors (Å^2^)				
Protein	157.06	120.76	164.21	59.3
Ligand	343.48	179.47	-	162.72
Root-mean-square deviations				
Bond lengths (Å)	0.003 (0)	0.005 (1)	0.003 (0)	0.005 (0)
Bond angles (°)	0.661 (2)	0.757 (11)	0.624 (12)	0.735 (5)
**Validation**				
MolProbity score	2.02	2	2.16	1.93
Clashscore	12.92	20.74	16.9	16.46
Poor rotamers (%)	0.48	0.79	0.28	0.5
Ramachandran plot				
Favored (%)	94.13	96.84	93.35	96.66
Allowed (%)	5.87	3.16	6.65	3.34
Disallowed (%)	0	0	0	0

	PRC2_AJ119__–__450_K4me3_ (EMD-43357, PDB 8VMI)	Nuc_H3K4me3_ (EMD-43358, PDB 8VMJ)	PRC2_AJ1__–__450_K4me3_ (EMD-43359, PDB 8VML)	Nuc_H3K4me3_ (EMD-43360, PDB 8VMN)
**Data collection and processing**				
Magnification	×81,000	×81,000	×81,000	×81,000
Voltage (kV)	300	300	300	300
Electron exposure (e^−^ per Å^2^)	50	50	50	50
Defocus range (μm)	−0.8 to −1.8	−0.8 to −1.8	−0.8 to −1.8	−0.8 to −1.8
Pixel size (Å)	0.525	0.525	0.43	0.43
Symmetry imposed	*C1*	*C1*	*C1*	*C1*
Initial particle images (no.)	11,241,197	11,241,197	5,701,235	5,701,235
Final particle images (no.)	108,372	108,372	102,257	102,257
Map resolution (Å)	3.1	3.1	3.5	3.5
FSC threshold	0.143	0.143	0.143	0.143
Map resolution range (Å)	2.5–6	2.6–4	2.9–6	3.0–5
**Refinement**				
Initial model used (PDB code)	6WKR	6WKR	6WKR	6WKR
Model resolution (Å)	3.2	3.3	4	3.76
FSC threshold	0.5	0.5	0.5	0.5
Map sharpening *B* factor (Å^2^)	78	74	82	86
Model composition				
Nonhydrogen atoms	14,939	12,540	14,947	12,540
Protein residues	1,945	776	1,992	776
Ligands	0	314	0	314
*B* factors (Å^2^)				
Protein	79	47.74	167.27	65.6
Ligand	–	125.6	–	135.68
Root-mean-square deviations				
Bond lengths (Å)	0.004 (0)	0.006 (2)	0.003 (2)	0.004 (0)
Bond angles (°)	0.691 (12)	0.742 (7)	0.659 (8)	0.666 (22)
**Validation**				
MolProbity score	1.95	1.6	1.7	1.59
Clashscore	11.11	12.26	6.41	12.03
Poor rotamers (%)	0.33	0.47	0.48	0.32
Ramachandran plot				
Favored (%)	94.27	98.03	94.98	98.16
Allowed (%)	5.73	1.97	5.02	1.84
Disallowed (%)	0	0	0	0

	PRC2_AJ119__–__450_, JARID2_107__–__121_, Nuc_unmodified_ (EMD-47133)	PRC2_AJ119__–__450_, JARID2_113__–__121, R115A_, Nuc_unmodified_ EMD-47135)
**Data collection and processing**		
Magnification	×36,000	×36,000
Voltage (kV)	200	200
Electron exposure (e^−^ per Å^2^)	50	50
Defocus range (μm)	−0.8 to −1.8	−0.8 to −1.8
Pixel size (Å)	0.57	0.57
Symmetry imposed	*C1*	*C1*
Initial particle images (no.)	5,367,549	3,632,252
Final particle images (no.)	73,778	32,084
Map resolution (Å)	3.8	6.8
FSC threshold	0.143	0.143
Map resolution range (Å)	2.2–12	6.0–18
**Refinement**		
Initial model used (PDB code)	–	–
Model resolution (Å)	–	–
FSC threshold	–	–
Map sharpening *B* factor (Å^2^)	–	–
Model composition		
Nonhydrogen atoms	–	–
Protein residues	–	–
Ligands	–	–
*B* factors (Å^2^)		
Protein	–	–
Ligand	–	–
Root-mean-square deviations		
Bond lengths (Å)	–	–
Bond angles (°)	–	–
**Validation**		
MolProbity score	–	–
Clashscore	–	–
Poor rotamers (%)	–	–
Ramachandran plot		
Favored (%)	–	–
Allowed (%)	–	–
Disallowed (%)	–	–

FSC, Fourier shell correlation.

In the tail-disengaged state, no density is seen extending from the histone core and interacting with the structural elements that channel it to the active site (Fig. 1d). There is residual density at the active site that most likely corresponds to a second JARID2 molecule (Extended Data Fig. 2e), as previously reported in structural work on JARID2-containing PRC2 complexes lacking histone substrates^6^. While we cannot totally discard the possibility that this density could correspond to the N-terminal residues of the histone H3 tail, this tail-disengaged state lacks any of the other contacts with the histone H3 tail that were previously observed in all other cryo-EM structures of PRC2 bound to nucleosome substrates. The tail-engaged state, on the other hand, closely resembles that of our previously published structure of PRC2 bound to a nucleosome containing an unmodified H3K36 (ref. ^5^), with continuous density for the H3 tail along the substrate-binding cavity (Extended Data Fig. 2f) and with H3K27 positioned for methylation at the active site (Extended Data Fig. 2g) despite the presence of the H3K36me3 modification. We found that, in such a state, H3K36me3 is accommodated by a displacement of the lysine side chain compared to our previous PRC2–nucleosome structure (Protein Data Bank (PDB) 6WKR)^5^ containing unmodified H3K36 (Fig. 1e). This small change preserves most contacts involving PRC2, the nucleosomal DNA and the histone H3 tail. The mixture of tail-engaged and tail-disengaged states observed for PRC2 when in the presence of the histone H3K36me3 modification suggests that this mark impacts the efficiency of PRC2 to engage the histone H3 tail. On the other hand, when the histone H3 tail is bound, as captured in the tail-engaged state, the H3K36me3 modification does not affect the binding of JARID2 to the EED regulatory site and the allosteric communication between the EED and EZH2 core subunits is not disrupted.

### H3K36me3 modifies the interaction between PRC2 and chromatin

**Fig. 2:**
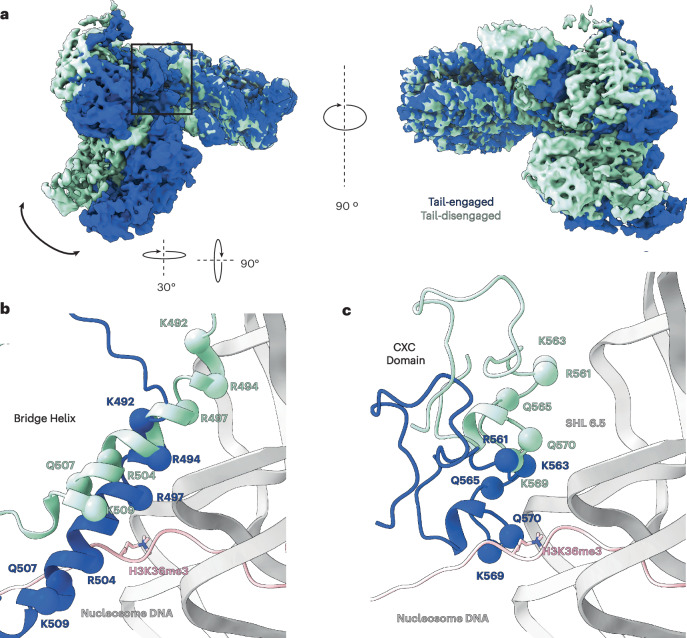
Comparison of tail-engaged and tail-disengaged PRC22_AJ1__–__450_ complexes bound to H3K36me3-modified nucleosomes. **a**, Overlay of the cryo-EM density maps for the coexisting tail-engaged (blue) and tail-disengaged (green) PRC2_AJ1__–__450_–H3K36me3 structures identified by our analysis. Maps are aligned using the nucleosome to show the relative rotation of PRC2 on the nucleosome surface. **b**, Close-up view of the EZH2 bridge helix showing its relative position with respect to the H3 tail (pink) and nucleosomal DNA (gray) for the tail-engaged (blue) and tail-disengaged (green) structures. **c**, Close-up view of the EZH2 CXC domain showing its relative position with respect to the H3 tail and nucleosomal DNA for the tail-engaged (blue) and tail-disengaged (green) structures (in **b**,**c**, the tail, as seen in the tail-engaged complex, is shown in pink).

Further comparison of the tail-engaged and tail-disengaged PRC2_AJ1–450_–H3K36me3 states revealed that the tail-disengaged complex is rotated with respect to the nucleosome interface by approximately 12° (Fig. 2a). This rotation results in a different DNA-binding register for both the EZH2 bridge helix (Fig. 2b) and the CXC domain (Fig. 2c) that offsets the bridge helix by approximately two helical turns (that is, seven residues). This seven-residue offset results in additional contacts between the bridge helix and nucleosomal DNA, involving residues that otherwise interact directly with the histone H3 tail in the tail-engaged state (Fig. 2b). Of notice, in the tail-engaged state, R504 interacts with the backbone of K36 in the histone H3 tail and R497 forms an electrostatic interaction with the backbone of the nucleosomal DNA (Extended Data Fig. 2f). In the tail-disengaged state, R504 satisfies the DNA contact made by R497 in the tail-engaged state, while R497 now makes an additional DNA contact upstream. Similarly, the binding geometry in the tail-disengaged state relocates bridge helix residue Q507 to a position where it can no longer interact with the H3 tail as is observed in the tail-engaged state. In addition, the CXC domain in the tail-disengaged state now contacts the major groove of the nucleosome DNA at superhelical location (SHL) 6.5 (while it contacts SHL 7 in the tail-engaged state) (Fig. 2c). Overall, despite this offset, both the residues involved and the nature of their interactions are maintained, consistent with the nonspecific nature of the CXC-mediated and bridge helix-mediated DNA interactions.

In previous structures of PRC2 engaged with nucleosome substrates, the carbonyl group of PRC2 Q570 in the CXC domain interacts with the ε-amino group of K36 in the histone tail^5,18^. The loss of hydrogen-bonding potential with the CXC domain when H3K36 is trimethylated most likely contributes to the alternative binding geometry that we observe in our cryo-EM data. Importantly, this alternative binding geometry in the presence of H3K36me3 reveals a unique interaction mode of PRC2 with chromatin that is incompatible with catalytic activity and highlights the sensitivity of the H3 tail-binding pocket within EZH2 to the surrounding chromatin environment.

### Variable PRC2–chromatin interaction in the absence of the H3 tail

To investigate how PRC2 engages with nucleosomes modified with H3K36me3 in the absence of allosteric activation, we omitted JARID2_K116me3_ from the sample preparation. A minimal construct of JARID2 containing residues 119–450 was shown to stabilize the PRC2 core, reduce dimerization of PRC2 and be essential for reaching high resolution in our previous cryo-EM studies of PRC2 complexes^6,17^. Therefore, we included this JARID2 construct in all structural efforts lacking JARID2_K116me3_ described in this paper. Under such conditions, PRC2 was shown to exhibit further reduced trimethylation activity on nucleosome substrates harboring H3K36me3 (Fig. 1b)^5,25^.

**Extended Data Fig. 3:**
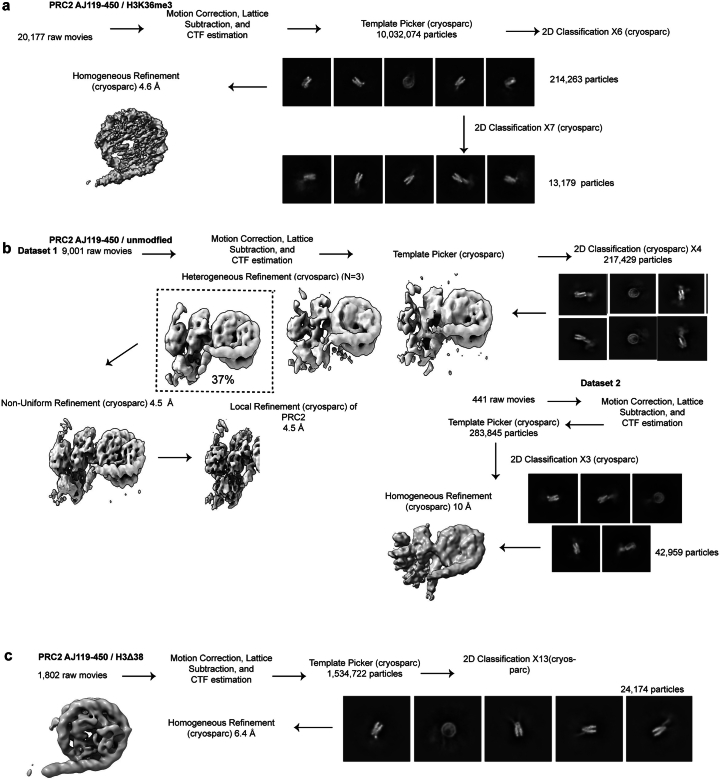
Cryo-EM of PRC2_AJ119-450_ bound to H3K36me3, unmodified, and H3Δ38 nucleosomes. **A)** Cryo-EM analysis of PRC2_AJ119-450_ bound to H3K36me3 modified nucleosomes. Data was extensively cleaned by 2D classification trying to bring up density for PRC2. Representative 2D classes after 6 rounds are shown, but the resulting 214,262 particles resulted in a 4.6 Å reconstruction showing only the nucleosome, without additional density for PRC2. Further cleaning of the data by 2D classification (after 13 total rounds) only showed fuzzy density for PRC2. **B)** Data collected for PRC2_AJ119-450_ bound to unmodified nucleosomes was processed in cryosparc following standard workflow. Particles were subjected to 2D classification, followed by heterogeneous refinement to remove damaged complexes. Non-uniform refinement was performed followed by local refinement around PRC2. A second dataset of 441 movies was collected and used to generate a reconstruction showing density of PRC2 for comparison with H3K36me3 and H3Δ38 complexes. **C)** Cryo-EM of PRC2_AJ119-450_ bound to nucleosomes containing H3Δ38. Data was extensively cleaned by 2D classification trying to bring up density for PRC2. Representative 2D classes after 13 rounds are shown. The resulting 24,172 particles were used to obtain a 6.4 Å reconstruction that shows only the nucleosome, without additional density for PRC2, despite the weak density that can be observed in the 2D classification.

Despite much effort, it was not possible to produce a structure of PRC2 lacking JARID2_K116me3_ bound to the H3K36me3 nucleosome because of the flexibility or heterogeneity of the engagement between them. Extensive two-dimensional (2D) classification of a large cryo-EM dataset showed only fuzzy density bound to nucleosomes (Extended Data Fig. 3a), suggesting that the positioning of PRC2 with respect to the nucleosome is variable (that is, there is no fixed register of PRC2 on the nucleosome) under these conditions. As a control, we could obtain reconstructions of the PRC2 complex lacking JARID2_K116me3_ when it was bound to unmodified nucleosomes from just a few hundred micrographs (Extended Data Fig. 3b). These results strongly suggest that the increased mobility observed at the PRC2–nucleosome interface is caused by the presence of the H3K36me3 modification and its negative effect on the engagement of PRC2 with substrate H3 tails.

**Extended Data Fig. 4:**
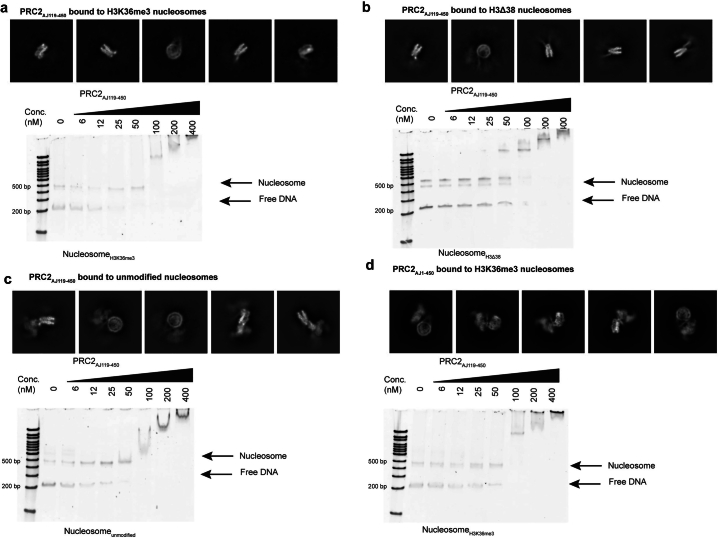
Cryo-EM and nucleosome binding analysis of PRC2 with H3K36me3, unmodified, and H3Δ38 nucleosomes. **A)** Representative 2D classes of PRC2_AJ119-450_ bound to H3K36me3 nucleosomes show absence of strong density for PRC2, despite observed binding in electromobility shift assays. Representative electromobility shift assays were performed with a titration of 0 to 400 nM PRC2 with 50 nM nucleosome in all cases. Results were reproduced in triplicate. **B)** Representative 2D classes of PRC2_AJ119-450_ bound to nucleosomes containing histone H3Δ38 show absence of strong density for PRC2, despite observed binding in electromobility shift assays. **C)** Representative 2D classes of PRC2_AJ119-450_ bound to unmodified nucleosomes that yielded the 4.5 Å reconstruction. **D)** Representative 2D classes of PRC2_AJ1-450_ bound to H3K36me3 nucleosomes that yielded the 3.6 Å tail-engaged reconstruction. In contrast to A and B, C and D show strong density for PRC2. Electromobility shift assays show similar binding affinity for PRC2_AJ119-450_ bound to unmodified nucleosomes or PRC2_AJ1-450_ bound to H3K36me3 nucleosomes to that observed for the PRC2_AJ119-450_/Nucleosome_H3K36me3_ and PRC2_AJ119-450_/Nucleosome_H3Δ38_ assays.

To assess how the histone H3 tail impacts the interaction of PRC2 with chromatin, we performed cryo-EM and electrophoretic mobility shift assays (EMSAs) of PRC2 complexes including or lacking JARID2_K116me3_ in the presence of H3Δ38-containing nucleosomes that lack the H3 tail residues that interact with EZH2 (Extended Data Fig. 3c) for comparison to those performed with unmodified or H3K36me3-containing nucleosomes. We found that PRC2 complexes bound to all three nucleosome substrates with similar affinity and independently of the presence of JARID2_K116me3_ (Extended Data Fig. 4a–d). Furthermore, cryo-EM analysis of PRC2_AJ119__–__450_ bound to nucleosomes lacking the histone H3 tail (N-terminally truncated at residue 38, H3Δ38) closely resembled that obtained for the H3K36me3-modified nucleosome (Extended Data Figs. 3c and 4b), further supporting the idea that, in the absence of H3 tail engagement with EZH2, the PRC2–nucleosome interaction is highly dynamic or variable and difficult to visualize by cryo-EM. These results suggest that although the histone H3 tail contributes minimally to the affinity of PRC2 for nucleosomes, which is dominated by electrostatic interactions with nucleosomal DNA^23,42^, the histone H3 tail is important for the functional engagement of PRC2 with chromatin. Several studies have suggested that targeting of PRC2 across the genome may be decoupled from its methyltransferase activity^12,43,44^. Our tail-disengaged PRC2–H3K36me3 state now shows a variable interaction of PRC2 with chromatin that is not productive for activity. This variability highlights one way in which PRC2 can engage chromatin without performing its catalytic function.

In summary, our results are consistent with a mechanism in which the histone H3K36me3 modification reduces productive engagement of the histone H3 tail with PRC2 and promotes dynamic interactions with chromatin that are incompatible with the trimethylation of histone H3K27 by PRC2 at actively transcribed gene bodies rich in this modification. This effect is partially overcome in the presence of JARID2_K116me3_, which interacts with both EED and EZH2 and thus stabilizes the catalytic lobe of the complex in a way that helps it retain the histone H3 tail in the active site.

### H3K4me3 binds to the EED allosteric site

**Fig. 3:**
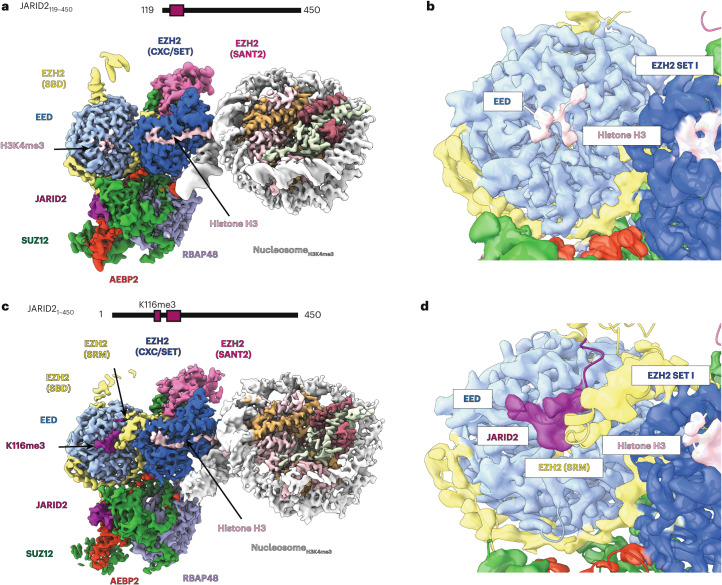
Cryo-EM structures of PRC2 bound to H3K4me3-modified nucleosomes. **a**, Cryo-EM structure of PRC2_AJ119__–__450_ bound to H3K4me3-modified nucleosomes. **b**, Close-up view of the cryo-EM density showing the N terminus of histone H3 containing H3K4me3 bound to EED. The map is shown at a threshold of 0.487 (lower threshold in Extended Data Fig. 7a). **c**, Cryo-EM structure of PRC2_AJ1__–__450_ bound to H3K4me3-modified nucleosomes. **d**, Close-up view of cryo-EM density showing JARID2_K116me3_ bound to EED and the folding of the SRM helix. The map is shown at a threshold of 0.25.

**Extended Data Fig. 5:**
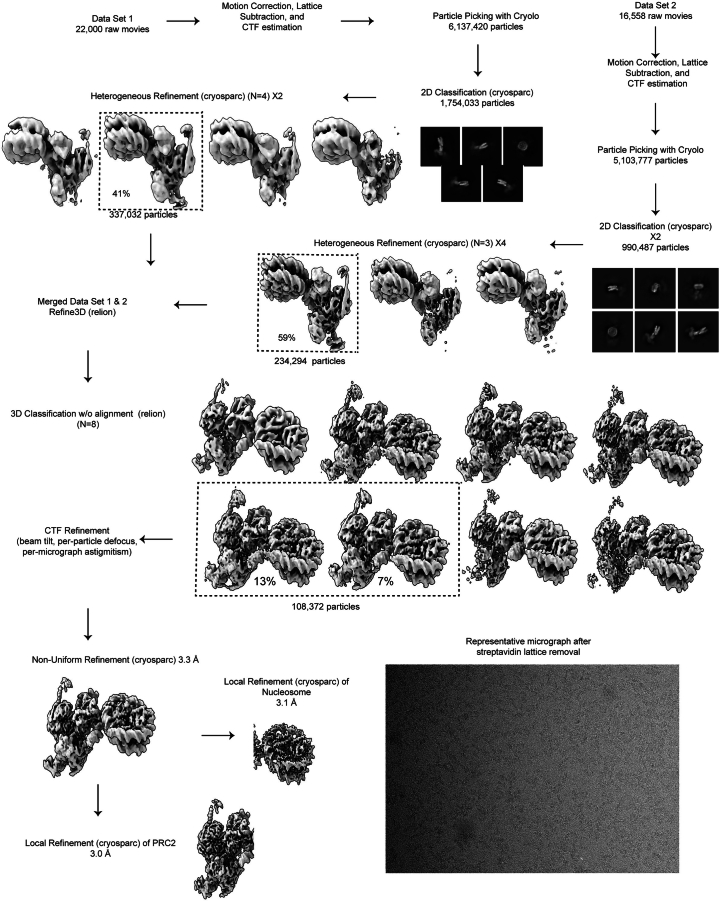
Processing workflow for PRC2_AJ119-450_ bound to H3K4me3 nucleosomes. Data collected for PRC2_AJ119-450_ bound to H3K4me3 nucleosomes was initially processed in cryosparc. Two data sets were merged after 2D classification and one round of heterogeneous refinement. Particles were imported into RELION for 3D Refinement followed by 3D classification without alignment. All 3D classes show density for the histone H3 tail and absence of density for the EZH2 SRM. Classes were selected showing the strongest density for H3K4me3 bound to EED. After CTF Refinement, particles were imported into cryosparc for non-uniform refinement and local refinements around the nucleosome and PRC2 regions. Source data

**Extended Data Fig. 6:**
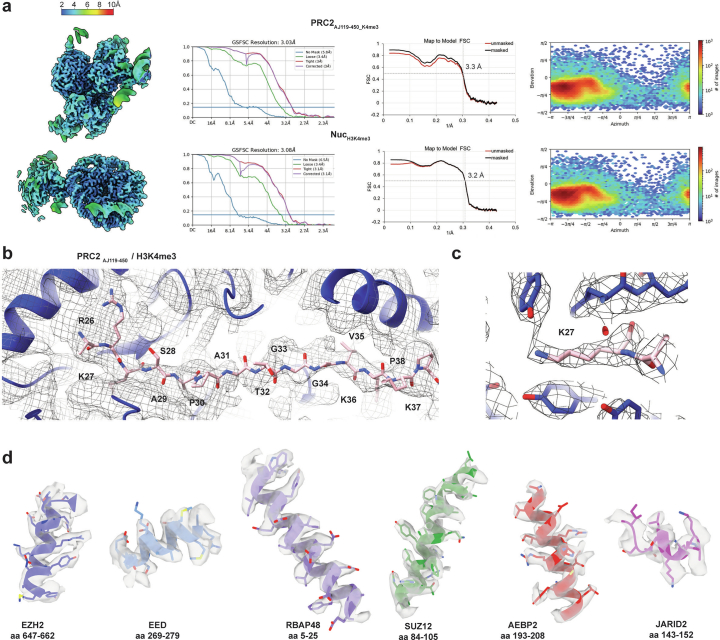
Local resolution maps for PRC2_AJ119-450_ complexes bound to H3K4me3-containing nucleosomes. **A)** Local resolution mapped onto cryo-EM maps of PRC2_AJ119-450_ complexes bound to H3K4me3-containing nucleosomes obtained from local refinements using masks surrounding PRC2 or nucleosome regions. For each map, the resolution at FSC = 0.143 is provided, while the map-to-model FSC plots show the masked resolution at FSC = 0.5. Each map has been locally filtered using the local resolution estimate. Angular distribution plot for particles that make up the final reconstructions are also included **B)** Examples of fitting of protein amino acid side chains into the density map for each subunit within the PRC2 complex. **C)** Cryo-EM density shown for the histone H3 tail (pink) bound to the EZH2 SET domain (blue). **D)** Close up view of histone H3K27 (pink) bound to the active site of EZH2 (blue).

**Extended Data Fig. 7:**
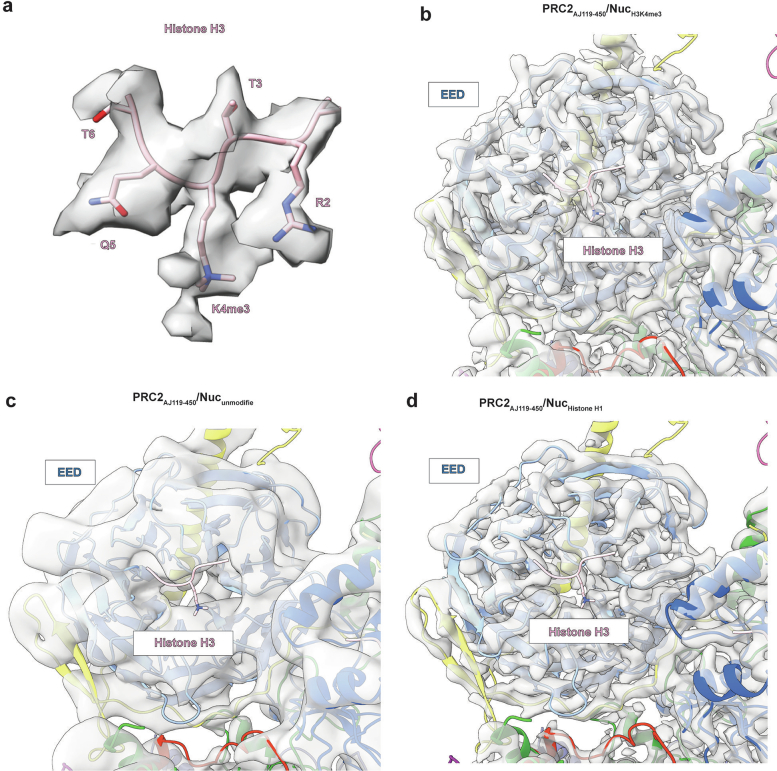
Comparison of cryo-EM density for the EED allosteric site in structures of PRC2_AJ119-450_ bound to H3K4me3 or to unmodified nucleosomes. **A)** Density surrounding the H3K4me3 bound to the EED for the PRC2_AJ119-450_ bound to a H3K4me3-modified nucleosome shown at threshold of 0.232 **B)** Cryo-EM density map (transparent grey) and model of the EED allosteric site and the bound methylated peptide for PRC2_AJ119-450_ bound to a H3K4me3-modified nucleosome. The model is shown in ribbon representation colored by subunit, with EED shown in light blue and the histone H3K4me3 peptide shown in pink. **C)** Corresponding view of a reconstruction obtained for PRC2_AJ119-450_ bound to a unmodified nucleosome showing lack of density in the EED allosteric pocket. **D)** Corresponding view of our recently reported reconstruction obtained for PRC2_AJ119-450_ bound to a an unmodified nucleosome that additionally contained the linker histone H1, also showing lack of density in the EED allosteric pocket.

Histone H3K4me3 localizes to actively transcribed promoters, where it has been demonstrated to interact directly with the transcription initiation machinery and recently shown to regulate promoter-proximal pausing^45–48^. In vitro biochemical assays showed that the H3K4me3 modification directly inhibits the activity of PRC2 but only when it is present in *cis* on nucleosome substrates^25^. To investigate how H3K4me3 inhibits PRC2 function, we obtained a 3-Å-resolution structure of PRC2 in the absence of JARID2_K116me3_ and bound to an H3K4me3-modified nucleosome (Table 1 and Extended Data Figs. 5 and 6a,b). Overall, this complex resembles other PRC2–nucleosome structures, in which the H3 tail is clearly visible entering the active site (Extended Data Fig. 6c) and H3K27 is positioned for catalysis (Extended Data Fig. 6d). Unlike the interactions with H3K36me3-modified nucleosomes, we were unable to identify a population of particles lacking tail engagement. Not surprisingly, given the absence of JARID2_K116me3_ as an allosteric activator, there is no density for the SRM and the SBD is in an extended conformation, both indicative of an unstimulated state (Fig. 3a). However, we do observe a methylated lysine bound to the aromatic cage of EED. We propose that this density corresponds to the H3K4me3 modification (Fig. 3b), the only source of methylated peptide in the sample. Indeed, the resolution of the PRC2_AJ119__–__450_–H3K4me3 structure allowed us to confidently build a peptide into the unassigned density with a sequence matching that of the N terminus of the H3 tail (Extended Data Fig. 7a,b) that agrees with a previous crystal structure of EED bound to an H3K4me3-containing peptide^49^. Although the affinity of EED for the H3K4me3 peptide was previously reported to be low, it is important to notice that our structure reports the interaction of H3K4me3 with EED in the context of the nucleosome, the relevant PRC2 substrate. To further eliminate the possibility that this peptide originated from the sample purification, we also determined the structure of PRC2_AJ119__–__450_ bound to an unmodified nucleosome substrate. In this structure (Extended Data Figs. 7c and 3b) and as in a previous reconstruction of PRC2–nucleosome also lacking JARID2_K116me3_ (ref. ^17^), the EED aromatic cage is vacant (Extended Data Fig. 7d). We, therefore, assigned the extra density in the PRC2_AJ119__–__450_–H3K4me3 structure to the H3 tail encompassing K4me3.

### JARID2 can compete with H3K4me3 for the EED allosteric site

**Extended Data Fig. 8:**
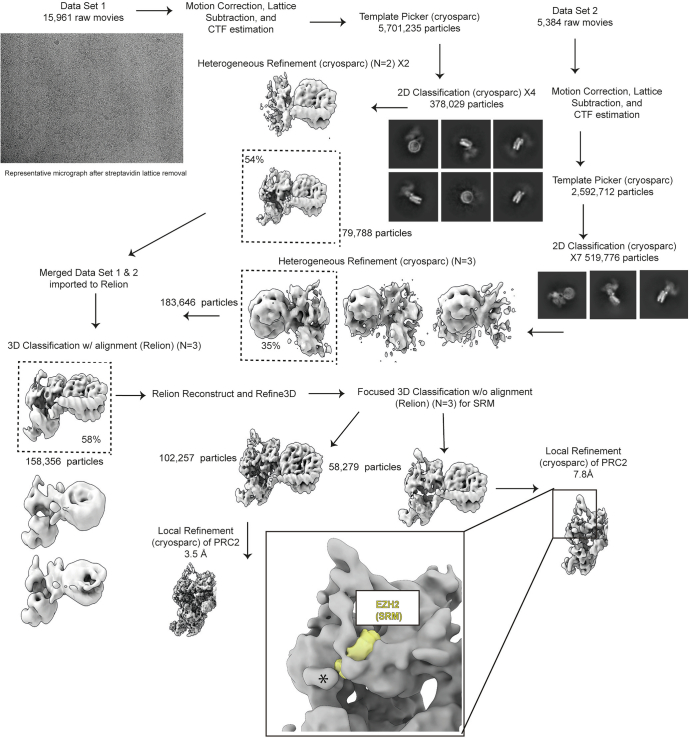
Processing workflow for PRC2_AJ1-450_ bound to H3K4me3 nucleosomes. Data collected for PRC2_AJ1-450_ bound to H3K4me3 nucleosomes was processed in cryosparc. Two data sets were merged after 2D classification and heterogeneous refinement. Particles were imported into RELION and subjected to 3D classification without alignment. Focused classification was performed using a mask around the allosteric site and EZH2 SRM regions resulting in two major classes showing the presence of absence of the SRM. Each class was refined separately and imported into cryosparc for local refinement around the PRC2 region. Close up view of the allosteric site of the PRC2_AJ1-450_ / H3K4me3 cryo-EM map (grey) showing the corresponding SRM density (yellow) in the ‘activated’ PRC2_AJ1-450_ / H3K4me3 cryo-EM map that has been lowpass filtered to to 8 Å. Both maps contain an unassigned density marked with asterisk that is not apparent in any of the other cryo-EM density maps of PRC2/nucleosome complexes.

**Extended Data Fig. 9:**
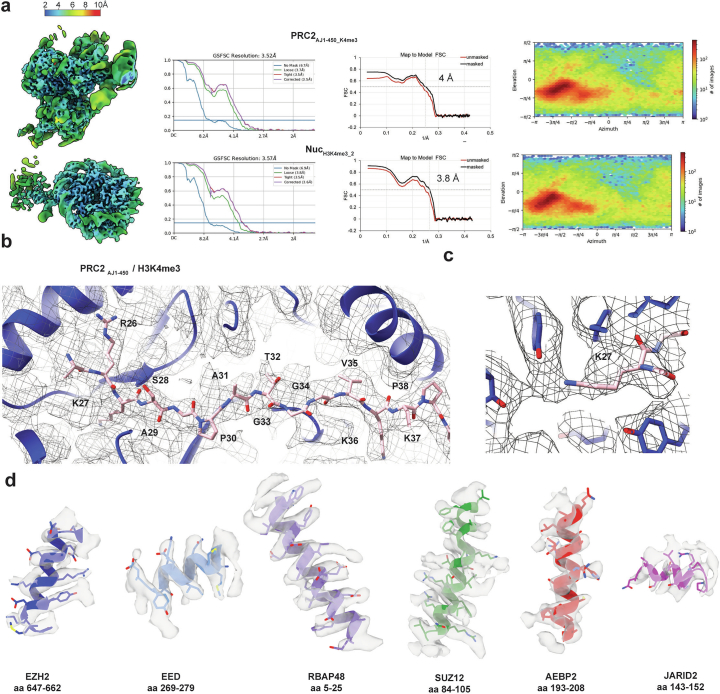
Local resolution maps for PRC2_AJ1-450_ complexes bound to H3K4me3-containing nucleosomes. **A)** Local resolution mapped onto cryo-EM maps of PRC2_AJ1-450_ complexes bound to H3K4me3-containing nucleosomes obtained from local refinements using masks surrounding PRC2 or nucleosome regions. For each map, the resolution at FSC = 0.143 is provided, while the map-to-model FSC plots show the masked resolution at FSC = 0.5. Each map has been locally filtered using the local resolution estimate. PRC2_AJ1-450_ / H3K4me3 **B)** Examples of fitting of protein amino acid side chains into the density map for each subunit within the PRC2 complex **C)** Cryo-EM density shown for the histone H3 tail (pink) bound to the EZH2 SET domain (blue). **D)** Close up view of histone H3K27 (pink) bound to the active site of EZH2 (blue).

Previous biochemical data showed that PRC2 can be activated on H3K4me3-modified nucleosome substrates in the presence of high local concentrations of allosteric activator (either excess H3K27me3 peptide or PRC2 complexes copurified with methylated JARID2)^5,25^. We, therefore, determined the structure of PRC2_AJ1__–__450_, thus including JARID2_K116me3_, bound to an H3K4me3-modified nucleosome at 3.5-Å resolution (Table 1 and Extended Data Figs. 8 and 9). In this structure, we observed an activated state of PRC2, with density consistent with JARID2_K116me3_ occupying the EED regulatory site, bent SBD and folded SRM (Fig. 3c,d). Importantly, we could not locate the histone H3K4me3 modification elsewhere in this reconstruction, supporting that JARID2_K116me3_ and H3K4me3 compete for the same binding site. Further, methyltransferase activity assays performed with PRC2_AJ1__–__450_ or with PRC2_AJ119__–__450_ in the presence of a methylated JARID2 peptide including residues 107–121 both displayed higher trimethylation of histone H3K27 compared to assays performed with PRC2_AJ119__–__450_ in the absence of activating peptide (Fig. 1b).

Although the activated state was prevalent in our data, careful sorting for occupancy of the SRM led us to identify a second, rarer state (resolution limited to 8 Å) in which the SRM is unfolded and the SBD is extended, although the EED regulatory site remains occupied (Extended Data Fig. 8). These features correspond to a nonactivated state of PRC2, similar to that obtained for the PRC2_AJ119__–__450_–H3K4me3 structure. The existence of both allosterically activated and inactivated PRC2 states in the cryo-EM data agrees with previous biochemical observations concerning activity under conditions including allosteric activators and suggests that JARID2 and H3K4me3 are mutually exclusive binders of the EED regulatory site.

### H3K4me3 acts as an allosteric antagonist

**Fig. 4:**
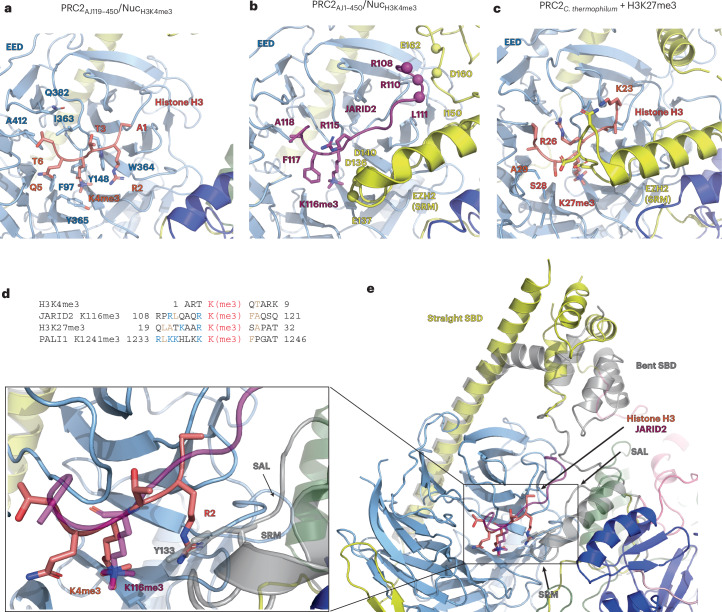
H3K4me3 acts as an allosteric antagonist by binding to EED. **a**, Interactions between the region of histone H3 (pink) around the H3K4me3 modification and the aromatic cage of EED (light blue). **b**, Interactions involving JARID2 (magenta), EED (light blue) and EZH2 (yellow for the SRM and SAL; darker blue for the SET domain) in an allosterically activated PRC2. The coordinates used were from the PRC2_AJ1__–__450_–H3K4me3 structure obtained in this study (Fig. 3c). JARID2 R115 interacts with E137 and D140 of the EZH2 SRM. JARID2 R108 and R110 are positioned to interact with E162 and D160 of EZH2, while JARID2 L111 and EZH2 I150 are involved in hydrophobic contacts. **c**, Interactions between the peptide around H3K27me3 (pink) and EED (light blue) and EZH2 (yellow for the SRM and SAL; darker blue for the SET domain). The coordinates used are those from the X-ray crystal structure of an activated PRC2 catalytic lobe from *Chaetomium thermophilum* (PDB 5KJH)^7^. Histone H3R26 (similarly to JARID2 R115) interacts with negatively charged residues in the EZH2 SRM and histone H3R23 establishes additional contacts with the SRM. **d**, Sequence alignment with respect to the PRC2-methylated lysine for the N-terminal region of the histone H3 tail around K4, JARID2 around K116, H3 around K27 and PALI1 around K1241. The PRC2-modified lysine is colored in red, residues that are involved in hydrophobic contacts are colored in tan and residues involved in electrostatic interactions are colored in blue. **e**, Overlay of structures shown in **a** (colored) and **b** (gray or transparent) showing that histone H3R2 of H3K4me3 clashes with the SAL, with the region corresponding to peptides bound to the allosteric site zoomed in on the right.

The recognition of methylated lysines by EED occurs through a classical hydrophobic cage. Previous studies indicated that sequence variation among EED-binding peptides is tolerated by subtle alternative binding modes involving residues within EED and EZH2 (refs. ^7,9,49^). Our study shows that histone H3K4me3 interacts with the aromatic cage in a similar manner to other EED-binding peptides and is further stabilized by interactions between histone residue H3T6 and the hydrophobic pocket defined by I363, Q382 and A412 of EED (Fig. 4a). To reconcile our finding that H3K4me3 binds to the EED regulatory site but fails to activate PRC2, we further compared its potential to interact with the EZH2 SRM with that of known allosteric activators of PRC2.

Our structures containing JARID2 K116me3 show that JARID2 is positioned to form several hydrophobic and electrostatic interactions with residues of the EZH2 SRM (Fig. 4b), as also described in previous cryo-EM and crystallographic studies of JARID2-containing PRC2 complexes^5,6,11^. Similar electrostatic interactions were also observed in a crystal structure of PRC2 bound to an H3K27me3-containing peptide (Fig. 4c) and biochemical perturbation experiments support the importance of these SRM residues for the stimulation of PRC2 activity^7,12^. Notably, the interactions with the EZH2 SRM created by JARID2 or H3K27me3 involve residues that are N terminal to the methylated lysine. Other activators, such as PALI1 K1241me3, contain sequence similarities at equivalent positions (specifically the positively charged residue in the −1 position), while these residues are not conserved or do not exist in H3K4me3 because of its location proximal to the N terminus of the histone H3 protein (Fig. 4d). Additionally, histone H3R2, the only positively charged residue that is N terminal to histone H3K4me3, sterically clashes with the SANT activation loop (SAL) in EZH2 (Fig. 4e) that is required for stimulation through EED^7^; as a consequence, density for the SAL is absent in our PRC2_AJ119_–H3K4me3 structure.

**Extended Data Fig. 10:**
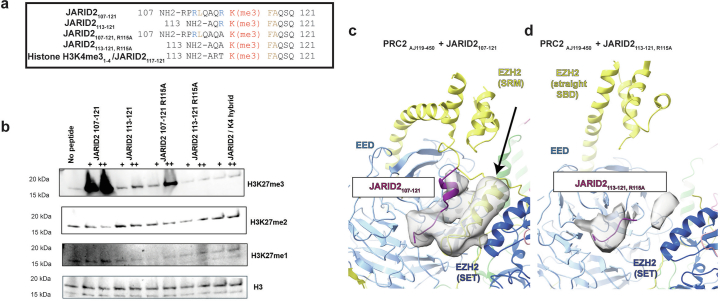
Activation of PRC2_AJ119-450_ by methylated JARID2 peptides. **A)** Methylated JARID2 peptide sequences used to perform methyltranserfase assays. **B)** Representative methyltransferase assays performed with PRC2_AJ119-450_ complexes on unmodified mononucleosome substrates in the absence or in the presence of 15 µM (+) or 150 µM (++) methylated JARID2 peptides. Results were reproduced in triplicate. **C)** Cryo-EM density for PRC2_AJ119-450_/unmodified nucleosome complex in the presence of JARID2_107-121_ shows strong density for the SRM helix. Map displayed after lowpass filtering to 7 Å. **D)** Cryo-EM density for PRC2_AJ119-450_/unmodified nucleosome complex in the presence of JARID2_113-121,R115A_ shows density corresponding to the peptide, but weak density for the SRM. Map displayed is lowpass filtered to 7 Å. Source data

To validate the importance of the N-terminal residues identified in allosteric activators, we performed methyltransferase assays with PRC2_AJ119__–__450_ complexes on unmodified nucleosome substrates in the presence of methylated JARID2 peptides (Extended Data Fig. 10a). Methylated JARID2 peptides including residues 107–121 were able to stimulate PRC2_AJ119__–__450_ to comparable levels to those of PRC2_AJ1__–__450_ complexes, which were copurified with methylated JARID2 (Fig. 1b). Substitution of R115 to alanine in the −1 position of JARID2 showed reduced H3K27me3 (Extended Data Fig. 10b), consistent with previous findings^11^. A JARID2 peptide lacking residues 107–112 in combination with substitution of R115 to alanine (JARID2_113_–_121,R115A_) was unable to stimulate PRC2 activity to levels beyond those of PRC2_AJ119__–__450_ complexes in the absence of JARID2 peptide. Consistent with these biochemical findings, cryo-EM data of PRC2_AJ119__–__450_ bound to nucleosomes in the presence of methylated JARID2_107__–__121_ but not in the presence of JARID2_113__–__121,R115A_ show PRC2 in an activated conformation with a folded SRM (Extended Data Fig. 10c,d).

Overall, the absence of contacts observed between residues around histone H3K4me3 and EZH2 explain why, in spite of its ability to engage with the aromatic cage of EED, there is a failure to stabilize the SRM for stimulation of EZH2 activity as seen for other EED binders that have been characterized as allosteric activators. An alternative (or additional) explanation that we are unable to exclude is that residues 7–23 of the histone H3 tail, which are invisible in our structure of PRC2_AJ119__–__450_–H3K4me3 (Fig. 3c), could themselves sterically block the folding of the SRM when H3K4me3 is engaged by the allosteric site. In conclusion, our structural analysis leads us to propose that the abundance of H3K4me3 localized on actively transcribed chromatin acts as allosteric antagonist to decrease PRC2 activity at these genomic locations.

## Discussion

**Fig. 5:**
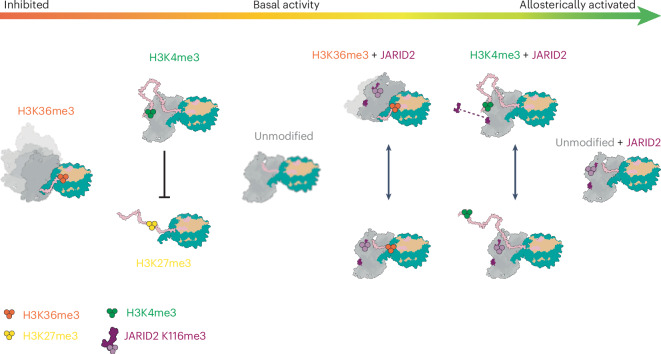
Model for the regulation of PRC2 catalytic activity by modifications of the H3 tail in the nucleosome substrate and the presence or absence of methylated JARID2. Trimethylation of histone H3K27 by PRC2 can be inhibited by H3K36me3 and H3K4me3 histone modifications and activated by H3K27me3 (in trans) or JARID2 K116me3. The interplay between these regulators is a gradient of PRC2 activity (red to green arrow). From left to right: in the absence of JARID2, H3K36me3 prevents efficient tail engagement and results in the loss of a well-defined register between PRC2 and the nucleosome substrate, depicted as the blurry model of PRC2; histone H3K4me3 engages the allosteric site and competes with nucleosomes that are already modified with H3K27me3; PRC2 on unmodified substrates in the absence of JARID2 has a basal level of activity, while its EED allosteric site remains open for possible engagement with nearby nucleosomes with H3K27me3; in the presence of JARID2, PRC2 is allosterically stimulated, resulting in increased H3K27me3 activity. Histone H3K36me3 and H3K4me3 reduce PRC2 activity through the same mechanisms described above; however, methylated JARID2 stabilizes the catalytic lobe of PRC2, facilitates some tail engagement in the presence of H3K36me3 and can compete with H3K4me3 for the EED allosteric site.

The combination of both positive and negative PRC2 regulatory mechanisms is required to fine-tune PRC2 activity to restrict the H3K27me3 repressive mark to specific genomic locations. This fine-tuning is achieved through the activation and inhibition of the complex through different mechanisms involving crosstalk between PRC2, its accessory subunits and existing marks on chromatin (Fig. 5). Both H3K4me3 and H3K36me3 are thought to serve as physical barriers for the spreading of H3K27me3. In support of this model, the depletion of either H3K4me3 or H3K36me3 methyltransferase machinery results in the redistribution of PRC2 across the genome and the invasion of H3K27me3 into domains that are decorated with H3K4me3 or H3K36me3 under normal conditions^36,50,51^.

In this study, we investigated the molecular mechanisms by which the histone PTMs H3K36me3 and H3K4me3, which are present in actively transcribed genes, directly inhibit the activity of PRC2. We found that H3K36me3 and H3K4me3 act through two distinct mechanisms because of their unique and distinct locations on the N-terminal histone H3 tail and target two important requirements for the higher-order methylation of histone H3K27: (1) the arrangement of PRC2, chromatin and the histone H3 tail that is conducive to efficient substrate engagement and (2) the activation of the catalytic SET domain through the EED–EZH2 regulatory axis. These two features permit the histone H3 tail to be retained in the active site for higher-order methylation states (dimethylation and trimethylation), which are the rate-limiting steps during PRC2 catalysis^52^. Both mechanisms of inhibition identified in this study provide potential avenues to break the autoactivating positive feedback loop established through EED–EZH2 and prevent the spreading of the H3K27me3 silencing mark into actively transcribed chromatin.

Histone H3K36 is adjacent to the nucleosome core particle, positioned at the entry site of the histone H3 tail in its path to the PRC2 active site. PRC2 interaction with nucleosomes involve two DNA-binding surfaces on EZH2, the bridge helix and the CXC domain, which directly interact with the unmodified H3K36 side chain^4,5,18^. Our structural data show that the trimethylation of H3K36 interferes with these interactions, resulting in the loss of a well-defined register for PRC2–nucleosome interactions that is required for effective H3 tail engagement and methylation. We propose that the proper, stable engagement of the histone H3 tail, although not the driver for the interaction between PRC2 and chromatin, is necessary to stabilize PRC2 on chromatin in a way that poises it for activity.

Histone H3K4, on the other hand, is located close to the N terminus of the flexible histone H3 tail, 23 residues upstream from the residue targeted by PRC2 (H3K27) and usually invisible in cryo-EM structures of PRC2 bound to nucleosome substrates. In our structure of PRC2 bound to H3K4me3-modified nucleosomes, we see that H3K4me3 can engage with the EED aromatic cage and occupy the allosteric site of EED. The EED–EZH2 allosteric activation mechanism has been studied extensively and is central to PRC2 function and its spreading of the H3K27me3 repressive mark^9^. The known EED binders are methylated peptides generated by the activity of EZH2 and include the accessory proteins JARID2 (in PRC2.2) and PALI1 (in PRC2.1), the histone H3K27me3 itself and, more recently identified, the EZH2 automethylation loop^9,13,17,53^. Because these methylated peptides take advantage of the same regulatory site, they are mutually exclusive and, therefore, each must be used in specific cellular contexts. Unlike H3K27me3, JARID2 K116me3 or PALI1 K1241me3, all of which allosterically stimulate PRC2, we show here that the engagement of H3K4me3 with EED is unique in that it fails to stabilize the SRM helix and stimulate PRC2 activity. At actively transcribed promoters, where chromatin is highly decorated with H3K4me3 and low in H3K27me3, the high local concentration of this mark may outcompete activators for the EED regulatory site, thus acting as an allosteric antagonist.

PRC2 is the sole writer in mammals of monomethylation, dimethylation and trimethylation of histone H3K27, with only the latter being associated with gene silencing^44^. Biochemical experiments have shown that both H3K4me3 and H3K36me3 have a minimal effect on the monomethylation of H3K27 (refs. ^18,25^) and, indeed, H3K27me1 is broadly found in the genome, including at actively transcribed regions^44,50,54^. Higher-order methylation, however, is prevented in those regions and appears to require both activation through the EED–EZH2 axis and stable engagement with the dynamic histone H3 tail. Dynamic expression levels of accessory PRC2 subunits such as JARID2 throughout development and across tissues may have a role in alleviating inhibition by H3K4me3 and H3K36me3 (ref. ^2^). For example, the increased expression of JARID2 may favor the engagement of the EED regulatory site with activating methyl-lysine-bearing peptides to start spreading the H3K27me3 repressive mark. In support of this model, the JARID2 cofactor is required during differentiation, when large sections of the genome require silencing, yet is dispensable in undifferentiated embryonic stem cells^13,55,56^. Additionally, our studies now show that the presence of stoichiometric, methylated JARID2 gives rise to stimulated PRC2 complexes engaged with H3K4me3-containing nucleosomes, which is consistent with in vitro biochemical experiments showing that PRC2 activity on H3K4me3-modified substrates can still be enhanced by JARID2 or H3K27me3 (refs. ^5,25^). Similarly, the presence of JARID2_K116me3_ also allowed us to obtain structures of stimulated PRC2 complexes bound to H3K36me3-modified substrates. The presence of methylated JARID2 in the EED resulted in the global stabilization of the catalytic lobe of PRC2 and enabled us to capture a dynamic state in which the H3K36me3-containing tail is accommodated by the subtle repositioning of the H3K36 side chain. A number of well-characterized roles in PRC2 regulation have already been assigned to JARID2: (1) residues 138–166 of JARID2 contribute to the stability to the PRC2.2 complex^6^; (2) JARID2_K116me3_ functions as a strong allosteric activator of PRC2 that can perform de novo H3K27me3 (refs. ^13,56^); and (3) the ubiquitin interaction motif of JARID2 recruits and stabilizes PRC2 on H2AK119Ub-modified chromatin^5,15^. Each of these activity-promoting functions could contribute to rescuing the inhibitory mechanisms imparted by either H3K4me3 or H3K36me3, thus facilitating the establishment of new heterochromatin domains.

The structures reported here provide mechanistic insight into the tight regulation of PRC2, involving a complex crosstalk between the PRC2 core, its accessory subunits, and the preexisting chromatin environment. Uncovering these and potential new mechanisms of regulation is essential for an understanding of how the Polycomb group proteins promote the correct gene silencing to safeguard developmental processes and to maintain cell identity.

## Methods

### Expression and purification of PRC2.2 complexes

PRC2 complexes containing AEBP2 and either JARID2_1__–__450_ or JARID2_119__–__450_ were cloned, expressed and purified as previously described^6^. Briefly, EZH2, EED, RBAP48, SUZ12 and Strep-tagged GFP fusions of AEBP2 and JARID2 were cloned into the Macrobac system for baculovirus expression in Sf9 insect cells. Cells were resuspended in lysis buffer (50 mM HEPES pH 7.9, 250 mM NaCl, 0.5 mM TCEP, 10% glycerol and 0.1% NP40) and supplemented with a EDTA-free protease inhibitor cocktail, leupeptin, pepstatin A, aprotinin and benzonase. Cells were lysed by sonication and cleared by centrifugation at 14,000*g* for 45 min. Supernatant was incubated with Strep-Tactin Superflow Plus resin overnight, washed with buffer containing 1 M NaCl, and eluted with 10 mM desthiobiotin. The eluate was subjected to tobacco etch virus protease cleavage followed by size-exclusion chromatography with a Superose 6 Increase 3.2/300 equilibrated with 50 mM HEPES pH 7.9, 150 mM KCl, 0.5 mM TCEP and 10% glycerol. The purified complex was flash-frozen in liquid nitrogen and stored at −80 °C as single-use aliquots.

### Nucleosome purification

For use in both cryo-EM and EMSA experiments, human nucleosomes containing unmodified H3, H3K4me3 or H3K36me3 were purchased from Epicypher with biotinylated DNA containing the following sequence:

GGACCCTATACGCGGCCGCCCTGGAGAATCCCGGTCTGCAGGCCGCTCAATTGGTCGTAGACAGCTCTAGCACCGCTTAAACGCACGTACGCGCTGTCCCCCGCGTTTTAACCGCCAAGGGGATTACTCCCTAGTCTCCAGGCACGTGTCAGATATATACATCCTGTGCCGGTCGCGAACAGCGACC-3′

Human octamers lacking the H3 tail were purchased from The Histone Source and reconstituted into nucleosomes by standard protocols. Biotinylated DNA containing the sequence ATATCTCGGGCTTATGTGATGGACCCTATACGCGGCCGCCCTGGAGAATCCCGGTGCCGAGGCCGCTCAATTGGTCGTAGACAGCTCTAGCACCGCTTAAACGCACGTACGCGCTGTCCCCCGCGTTTTAACCGCCAAGGGGATTACTCCCTAGTCTCCAGGCACGTGTCAGATATATACATCCTGTGCATGTATTGAACAGCGACTCGGGATAT was amplified by PCR and purified over a monoQ column with ethanol precipitation before being resuspended in high-salt buffer (10 mM Tris-HCl pH 7.5, 2 M KCl, 1 mM EDTA and 1 mM DTT). Then, 4.4 μM octamer was mixed with 4 μM biotinylated DNA and subjected to slow dialysis over 16 h into low-salt buffer (10 mM Tris-HCl pH 7.5, 250 mM KCl, 1 mM EDTA and 1 mM DTT) before being dialyzed into nucleosome storage buffer (20 mM Tris-HCl pH 7.5, 1 mM EDTA and 1 mM DTT).

*Xenopus* nucleosomes used for activity assays, either unmodified or modified with H3K4me3 or H3K36me3, were assembled using standard procedures. The methyl analogs H3K4cme3 and H3K36cme3 were purchased from The Histone Source. Histones H3 and H4 were resuspended in histone unfolding buffer (6 M GuHCl, 20 mM Tris-HCl pH 7.5 and 5 mM DTT), combined at an equimolar ratio and dialyzed into refolding buffer (10 mM Tris-HCl pH 7.5, 2 M NaCl, 1 mM EDTA and 5 mM β-mercaptoethanol). Refolded H3–H4 tetramers were then combined with purified soluble H2A–H2B dimers at an equimolar ratio and purified over a Superdex 200 10/300 GL column. Nucleosomes were assembled following the procedure described above.

### Cryo-EM grid preparation

All cryo-EM samples were prepared using streptavidin affinity grids that were fabricated in-house as previously described^40,41^. PRC2–nucleosome complexes were assembled by incubating 100 nM nucleosome with 500 nM PRC2 in cryo buffer (50 mM HEPES pH 7.5, 50 mM KCl, 0.5 mM TCEP and 100 μM *S*-adenosyl homocysteine). The sample was applied to rehydrated streptavidin affinity grids and incubated for 3–5 min at room temperature in a humidity chamber. Following incubation, grids were washed with freezing buffer (50 mM HEPES pH 7.5, 50 mM KCl, 0.5 mM TCEP, 4% trehalose and 0.01% NP40). Excess buffer was manually blotted away and 4 μl of freezing buffer was applied before transferring grids to the Leica GP2 automated plunger. Grids were blotted for 4–5 s using the Leica GP2 blot sensor before plunging into liquid ethane.

### Data collection and processing

High-resolution datasets were collected on a Titan Krios G3i microscope equipped with a Gatan Quantum energy filter (slit width 20 eV) at the University of California, Berkeley QB3 Cal-Cryo facility or at the Stanford Linear Accelerator Center Cryo-EM Center (S2C2).

Videos from all datasets were motion-corrected and dose-weighted using MotionCor2 (ref. ^57^) and then the streptavidin lattice was removed from the images using MATLAB^40^. Contrast transfer function (CTF) estimation was performed using CTFfind4^58^.

To obtain the reconstruction of PRC2_AJ119__–__450_ bound to H3K4me3-modified nucleosomes, 22,000 raw videos of 50 frames were collected for dataset 1 and 16,585 raw videos were collected for dataset 2. Both datasets were collected using super-resolution with a pixel size of 0.525 Å, a total dose of 50 e^−^ per Å^2^ and a defocus range between −0.8 and −1.8 μm. A total of ~6 million particles were picked for each dataset using a trained convolutional neural network in CRYOLO^59^. Initial 2D classification and three-dimensional (3D) heterogeneous refinement were performed in CryoSPARC^58^. Both datasets were merged and imported into RELION^60^ for 3D classification without alignment. CTF refinement was performed to correct for beam tilt, per-particle defocus and per-micrograph astigmatism. CTF-refined particles were imported into CryoSPARC for local refinement. Maps were filtered by local resolution using manually adjusted *B* factors to prevent oversharpening and the resulting density map was used for modeling.

For PRC2_AJ1__–__450_ bound to H3K4me3-modified nucleosomes, 15,961 raw videos were collected using super-resolution with a pixel size of 0.43 Å. A total of ~5.6 million particles were picked using template picker in CryoSPARC with 2D classes generated from a smaller subset of particles picked with the CryoSPARC blob picker. Initial 2D classification and 3D heterogeneous refinement were performed in CryoSPARC. Particles were imported into RELION for 3D classification without alignment. Particles were then subjected to focused classification around the EED and SRM region to identify a subset of particles lacking density for the SRM.

To obtain the reconstruction of PRC2_AJ1__–__450_ bound to H3K36me3-modified nucleosomes, 14,796 raw videos of 50 frames were collected with a super-resolution pixel size of 0.525 Å, a total dose of 50 e^−^ per Å^2^ and a defocus range between −0.8 and −1.8 μm. A total of ~4.3 million particles were picked for each dataset using a trained convolutional neural network in CRYOLO^59^. Initial 2D classification and 3D classification was performed in RELION. Focused classification was performed around the nucleosome and EZH2 SET domain to sort for a state that had density for the histone H3 tail. Focused classification was then performed on the PRC2 top lobe. The tail-disengaged class of particles was obtained through heterogeneous refinement in CryoSPARC, selecting for particles lacking density for the histone H3 tail. CTF refinement was performed for beam tilt, per-particle defocus and per-micrograph astigmatism for tail-engaged and tail-disengaged particle stacks. CTF-refined particles were imported into CryoSPARC for nonuniform and local refinements.

For PRC2_AJ119__–__450_ bound to unmodified nucleosomes, 9,001 raw videos were collected in super-resolution with a pixel size of 0.43 Å. Particles were picked using template picker in CryoSPARC and subjected to several rounds of 2D classification and heterogeneous refinement.

For PRC2_AJ119__–__450_ bound to H3K36me3-modified nucleosomes, 21,177 raw videos were collected in super-resolution with a pixel size of 0.43 Å. Particles were picked using template picker in CryoSPARC and subjected to many rounds of 2D classification to bring out density for PRC2. Only after 11 rounds of 2D classification could PRC2 be observed in the sample. Ab initio reconstruction failed, likely because of lack of views that were sorted out in the extensive cleaning of the data by 2D classification. A second dataset of 1,331 videos was collected on a Talos Arctica with super-resolution and a pixel size of 0.57 Å, a total dose of 50 e^−^ per Å^2^ and a defocus range between −0.8 and −1.8 μm. Particles were subjected again to extensive 2D classification to bring out density for PRC2. An ab initio reconstruction was obtained but PRC2 could not be resolved. On the same day, 441 videos were collected for PRC2_AJ119__–__450_ bound to unmodified nucleosomes. Particles were picked using template picker in CryoSPARC. Then 2D classification and ab initio reconstruction were performed in CryoSPARC and a low-resolution map was obtained containing density for both the unmodified nucleosome and PRC2.

For the sample containing PRC2_AJ119__–__450_ bound to H3Δ38 nucleosomes, 1,807 raw videos were collected on a Talos Arctica in super-resolution with a pixel size of 0.57 Å, a total dose of 50 e^−^ per Å^2^ and a defocus range between −0.8 and −1.8 μm. Particles were subjected to extensive 2D classification to bring out density for PRC2. An ab initio reconstruction was obtained but PRC2 could not be resolved.

### Model building

For all PRC2–nucleosome structures obtained in this study, we used our previously reported structure of PRC2 bound to an ubiquitylated nucleosome (PDB 6WKR)^5^ as the initial model. Coordinates were adjusted using flexible fitting in Isolde (version 1.5)^61^ in UCSF ChimeraX (version 1.5)^62^ and Coot^63^. Models were then iteratively refined and adjusted using PHENIX^64^ and Coot.

### EMSA

The PRC2–nucleosome reactions were prepared varying the concentration of PRC2 between 0 and 400 nM with 50 nM of unmodified, H3K4me3-modified, H3K36me3-modified or H3Δ38 nucleosomes in cryo buffer (50 mM HEPES pH 7.5, 50 mM KCl and 0.5 mM TCEP). Reactions were incubated for 5 min at room temperature. Glycerol was added to the reaction to a final concentration of 5% just before samples were loaded onto a 5% native TBE gel in 0.5× TBE buffer. Gels were stained with SYBR gold.

### Histone methyltransferase assays

Assays were carried out in a total volume of 12 µl with 750 nM *Xenopus* nucleosomes that were either unmodified of modified with H3K4me3 or H3K36me3 and 1.5 µM PRC2 in reaction buffer (50 mM HEPES pH 7.9, 50 mM KCl, 2.5 mM MgCl_2_, 0.25 mM EDTA, 0.5 mM TCEP and 100 µM *S*-adenosyl methionine). The JARID2 peptides used were synthesized by Synpeptide and used at concentrations of 15 or 150 µM when indicated. Reactions were incubated for 90 min at room temperature and quenched with 4× SDS loading dye and heat inactivation at 95 °C for 5 min before separation by gel electrophoresis. Gels were transferred to 0.2 µM PVDF using a Trans-blot Turbo system at 25 V for 5 min. The membranes were probed with antibodies to H3K27me1 (Cell Signaling, 84932), H3K27me2 (Cell Signaling, 9728), H3K27me3 (Cell Signaling, 9733) and histone H3 (Abcam, ab1791).

### Reporting summary

Further information on research design is available in the Nature Portfolio Reporting Summary linked to this article.

## Online content

Any methods, additional references, Nature Portfolio reporting summaries, source data, extended data, supplementary information, acknowledgements, peer review information; details of author contributions and competing interests; and statements of data and code availability are available at 10.1038/s41594-024-01452-x.

## Supplementary information

Reporting Summary

## Source data

Source Data Fig. 1 and Extended Data Fig. 10 Unprocessed western blots.

Source Data Extended Data Fig. 5 Unprocessed native PAGE gels used for EMSAs.

## Data Availability

Cryo-EM maps and fitted models were deposited to the EM Data Bank (under accession numbers EMD-43361, EMD-43373, EMD-43362, EMD-43363, EMD-43357, EMD-43358, EMD-43359, EMD-43360, EMD-47133 and EMD-47135) and the PDB (under accession numbers 8VNV, 8VOB, 8VNZ, 8VO0, 8VMI, 8VMJ, 8VML and 8VMN). Corresponding accession codes for each structure can be found in Table 1. Source data are provided with this paper.
