# Supplementary material for: Structural basis for the inhibition of PRC2 by active transcription histone posttranslational modifications
Source: Nat Struct Mol Biol. 2025 Jan 7;32(2):393–404. doi: 10.1038/s41594-024-01452-x (PMC11832421; doi:10.1038/s41594-024-01452-x)
Supplement: Supplementary file 1 — Reporting Summary [file 41594_2024_1452_MOESM1_ESM.pdf]

nature portfolio

Corresponding author(s): Eva Nogales

Last updated by author(s): Oct 3, 2024

Reporting Summary

Nature Portfolio wishes to improve the reproducibility of the work that we publish. This form provides structure for consistency and transparency in reporting. For further information on Nature Portfolio policies, see our Editorial Policies and the Editorial Policy Checklist.

Statistics

For all statistical analyses, confirm that the following items are present in the figure legend, table legend, main text, or Methods section.

n/a	Confirmed	
☐	☒	The exact sample size (*n*) for each experimental group/condition, given as a discrete number and unit of measurement
☐	☒	A statement on whether measurements were taken from distinct samples or whether the same sample was measured repeatedly
☒	☐	The statistical test(s) used AND whether they are one- or two-sided<br>*Only common tests should be described solely by name; describe more complex techniques in the Methods section.*
☒	☐	A description of all covariates tested
☒	☐	A description of any assumptions or corrections, such as tests of normality and adjustment for multiple comparisons
☒	☐	A full description of the statistical parameters including central tendency (e.g. means) or other basic estimates (e.g. regression coefficient) AND variation (e.g. standard deviation) or associated estimates of uncertainty (e.g. confidence intervals)
☒	☐	For null hypothesis testing, the test statistic (e.g. *F*, *t*, *r*) with confidence intervals, effect sizes, degrees of freedom and *P* value noted<br>*Give P values as exact values whenever suitable.*
☒	☐	For Bayesian analysis, information on the choice of priors and Markov chain Monte Carlo settings
☒	☐	For hierarchical and complex designs, identification of the appropriate level for tests and full reporting of outcomes
☒	☐	Estimates of effect sizes (e.g. Cohen's *d*, Pearson's *r*), indicating how they were calculated

Our web collection on statistics for biologists contains articles on many of the points above.

Software and code

Policy information about availability of computer code

Data collection	Serial EM 4-0-20 was used for cryo-EM data collection at Cal-Cryo and EPU 2.1 for cryo-EM data collection at Stanford SLAC Facility
Data analysis	Matlab2021b, Relion 3.1, Cryosparc 3.1, PHENIX 1.19.2, COOT 0.95, CRYOLO 1.7.6, ChimeraX 1.5, Pymol 2.5

For manuscripts utilizing custom algorithms or software that are central to the research but not yet described in published literature, software must be made available to editors and reviewers. We strongly encourage code deposition in a community repository (e.g. GitHub). See the Nature Portfolio guidelines for submitting code & software for further information.

Data

Policy information about availability of data

All manuscripts must include a data availability statement. This statement should provide the following information, where applicable:

- Accession codes, unique identifiers, or web links for publicly available datasets
- A description of any restrictions on data availability
- For clinical datasets or third party data, please ensure that the statement adheres to our policy

Cryo-EM maps and fitted models have been deposited in the Electron Microscopy Data Bank (EMDB) and the Protein Data Bank (PDB) under the accession numbers EMD-43361 EMD-43373 EMD-43362 EMD-43363 EMD-43357 EMD-43358 EMD-43359 EMD-43360 47133 EMD-47135 and PDB 8VNV 8VOB 8VNZ 8VO0 8VMI 8VMJ 8VML 8VMN. Corresponding accession codes for each structure can be found in Table 1.

Research involving human participants, their data, or biological material

Policy information about studies with human participants or human data. See also policy information about sex, gender (identity/presentation), and sexual orientation and race, ethnicity and racism.

Reporting on sex and gender	N/A
Reporting on race, ethnicity, or other socially relevant groupings	N/A
Population characteristics	N/A
Recruitment	N/A
Ethics oversight	N/A

Note that full information on the approval of the study protocol must also be provided in the manuscript.

Field-specific reporting

Please select the one below that is the best fit for your research. If you are not sure, read the appropriate sections before making your selection.

☒ Life sciences ☐ Behavioural & social sciences ☐ Ecological, evolutionary & environmental sciences

For a reference copy of the document with all sections, see nature.com/documents/nr-reporting-summary-flat.pdf

Life sciences study design

All studies must disclose on these points even when the disclosure is negative.

Sample size	Number of particles are specified in the Methods section and data processing workflow figures for each cryo-EM data set in this manuscript.
Data exclusions	Particles that were excluded from the final map through cryo-EM data processing are described in the Methods section of the manuscript.
Replication	Cryo-EM data collections were not repeated which is standard in the field, with the exception of PRC2_AJ119/H3K36me3 which was repeated twice. All electrommobility shift assays and western blot methyltransferase activity assays were replicated a minimum of three times.
Randomization	Following resolution estimation by the "gold standard" FSC method, particles sets were randomly assigned to two half sets.
Blinding	Blinding is not relevant for cryo-EM studies.

Reporting for specific materials, systems and methods

We require information from authors about some types of materials, experimental systems and methods used in many studies. Here, indicate whether each material, system or method listed is relevant to your study. If you are not sure if a list item applies to your research, read the appropriate section before selecting a response.

Materials & experimental systems

n/a	Involved in the study	
☐	☒	Antibodies
☐	☒	Eukaryotic cell lines
☒	☐	Palaeontology and archaeology
☒	☐	Animals and other organisms
☒	☐	Clinical data
☒	☐	Dual use research of concern
☒	☐	Plants

Methods

n/a	Involved in the study	
☒	☐	ChIP-seq
☒	☐	Flow cytometry
☒	☐	MRI-based neuroimaging

Antibodies

Antibodies used	H3K27me1 (Cell Signaling #84932, 1:1000 dilution) , H3K27me2 (Cell Signaling #9728, 1:1000 dilution), H3K27me3 (Cell Signaling #9733, 1:1000 dilution), and histone H3 (Abcam ab1791, 1:5000 dilution) were used for methyltransferase assays.

Validation	Mono-Methyl-Histone H3 (Lys27) (D3R8N) Rabbit mAb #84932 https://www.cellsignal.com/products/primary-antibodies/mono-methyl-histone-h3-lys27-d3r8n-rabbit-mab/84932?srsltid=AfmBOook9eiRg7CBYajTFPAy0iQo02yvP5c61Gc4JYsxefoDUQNR7lAx<br>Di-Methyl-Histone H3 (Lys27) (D18C8) XP® Rabbit mAb #9728 https://www.cellsignal.com/products/primary-antibodies/di-methyl-histone-h3-lys27-d18c8-xp-rabbit-mab/9728<br>Tri-Methyl-Histone H3 (Lys27) (C36B11) Rabbit mAb #9733 https://www.cellsignal.com/products/primary-antibodies/tri-methyl-histone-h3-lys27-c36b11-rabbit-mab/9733?srsltid=AfmBOoojamCq-r_KlrTKALDjyH4fN6ORQ6FHQf2sYIX9dlgMgqd9gI56<br>Anti-Histone H3 antibody - Nuclear Marker and ChIP Grade (abcam ab1791) https://www.abcam.com/en-us/products/primary-antibodies/histone-h3-antibody-nuclear-marker-and-chip-grade-ab1791

Eukaryotic cell lines

Policy information about cell lines and Sex and Gender in Research

Cell line source(s)	Sf9 cells were used for baculovirus production and protein expression. Cells were purchased from the University of California, Berkeley Cell Culture Facility.
Authentication	No further authentication procedures were used. Sf9 cells were monitored for appropriate doubling time, morphology, and confirmed to be devoid of contamination.
Mycoplasma contamination	Cells tested negative for mycoplasma contamination.
Commonly misidentified lines (See ICLAC register)	No misidentified lines were used in this work.

Plants

Seed stocks	N/A
Novel plant genotypes	N/A
Authentication	N/A
