# Supplementary material for: Structural basis for the inhibition of PRC2 by active transcription histone posttranslational modifications
Source: Nat Struct Mol Biol. 2025 Jan 7;32(2):393–404. doi: 10.1038/s41594-024-01452-x (PMC11832421; doi:10.1038/s41594-024-01452-x)

Figure 1B

Poncea S.

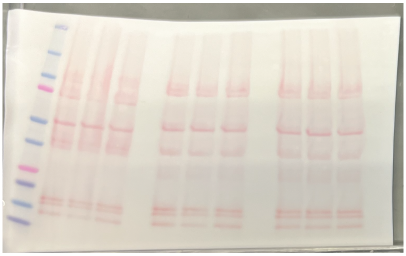

anti-H3

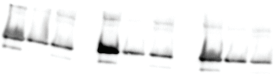

anti-H3K27me1

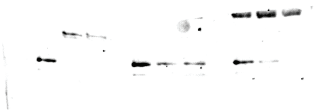

anti-H3K27me2

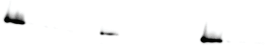

anti-H3K27me3

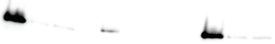

Extended Figure 13D

Poncea S.

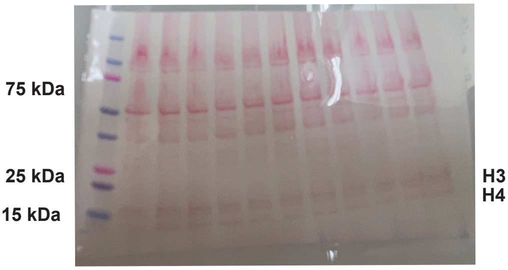

anti-H3

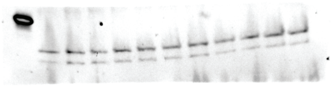

anti-H3K27me1

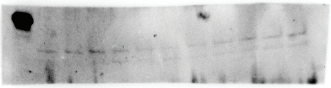

anti-H3K27me2

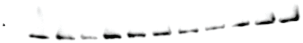

anti-H3K27me3

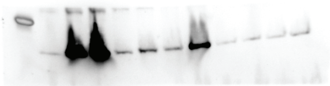

Supplement: Supplementary file 2 — Unprocessed western blots. [file 41594_2024_1452_MOESM2_ESM.pdf]
