# Supplementary figures and images for: Structural basis for the inhibition of PRC2 by active transcription histone posttranslational modifications
Source: Nat Struct Mol Biol. 2025 Jan 7;32(2):393–404. doi: 10.1038/s41594-024-01452-x (PMC11832421; doi:10.1038/s41594-024-01452-x)

**Extended Figure 5a**

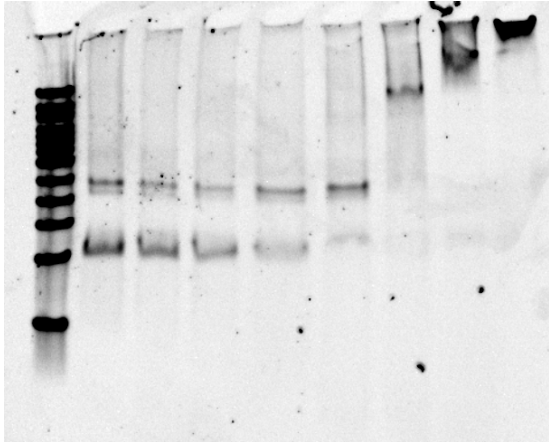

**Extended Figure 5b**

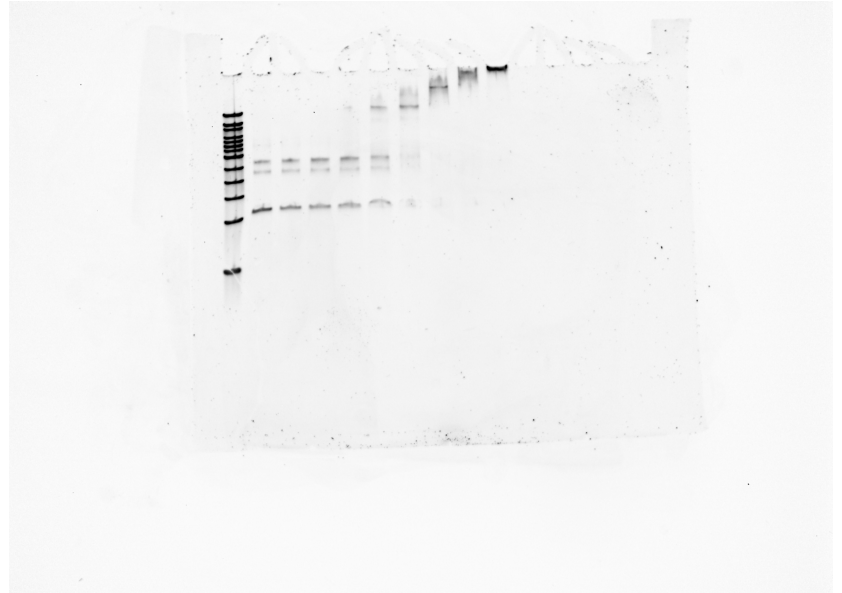

**Extended Figure 5c**

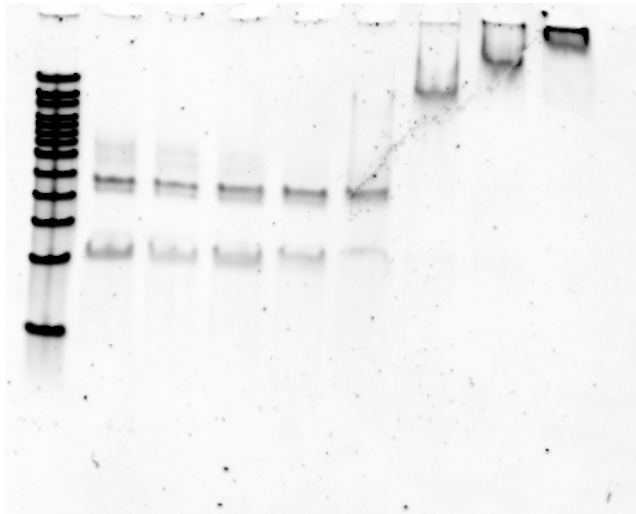

**Extended Figure 5d**

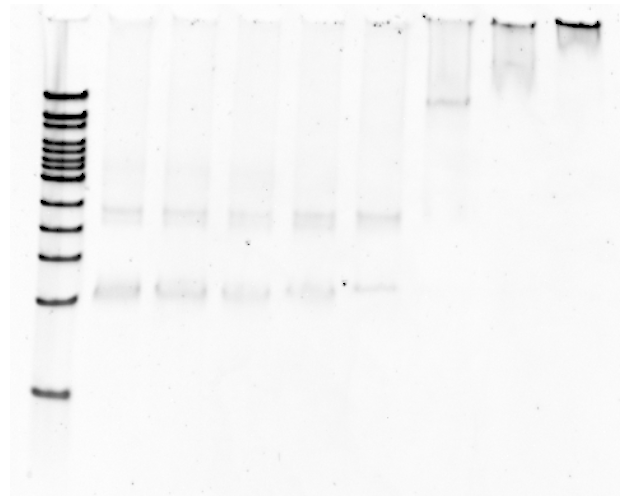

Supplement: Supplementary file 3 — Unprocessed native PAGE gels used for EMSAs. [file 41594_2024_1452_MOESM3_ESM.pdf]
